# Predicting Unplanned Readmissions Following a Hip or Knee Arthroplasty: Retrospective Observational Study

**DOI:** 10.2196/19761

**Published:** 2020-11-27

**Authors:** Ramin Mohammadi, Sarthak Jain, Amir T Namin, Melissa Scholem Heller, Ramya Palacholla, Sagar Kamarthi, Byron Wallace

**Affiliations:** 1 Northeastern University Boston, MA United States; 2 Tufts University School of Medicine Boston, MA United States

**Keywords:** deep learning, natural language processing, electronic health records, auto ML, 30-days readmission, hip arthroplasty, knee arthroplasty

## Abstract

**Background:**

Total joint replacements are high-volume and high-cost procedures that should be monitored for cost and quality control. Models that can identify patients at high risk of readmission might help reduce costs by suggesting who should be enrolled in preventive care programs. Previous models for risk prediction have relied on structured data of patients rather than clinical notes in electronic health records (EHRs). The former approach requires manual feature extraction by domain experts, which may limit the applicability of these models.

**Objective:**

This study aims to develop and evaluate a machine learning model for predicting the risk of 30-day readmission following knee and hip arthroplasty procedures. The input data for these models come from raw EHRs. We empirically demonstrate that unstructured free-text notes contain a reasonably predictive signal for this task.

**Methods:**

We performed a retrospective analysis of data from 7174 patients at Partners Healthcare collected between 2006 and 2016. These data were split into train, validation, and test sets. These data sets were used to build, validate, and test models to predict unplanned readmission within 30 days of hospital discharge. The proposed models made predictions on the basis of clinical notes, obviating the need for performing manual feature extraction by domain and machine learning experts. The notes that served as model inputs were written by physicians, nurses, pathologists, and others who diagnose and treat patients and may have their own predictions, even if these are not recorded.

**Results:**

The proposed models output readmission risk scores (propensities) for each patient. The best models (as selected on a development set) yielded an area under the receiver operating characteristic curve of 0.846 (95% CI 82.75-87.11) for hip and 0.822 (95% CI 80.94-86.22) for knee surgery, indicating reasonable discriminative ability.

**Conclusions:**

Machine learning models can predict which patients are at a high risk of readmission within 30 days following hip and knee arthroplasty procedures on the basis of notes in EHRs with reasonable discriminative power. Following further validation and empirical demonstration that the models realize predictive performance above that which clinical judgment may provide, such models may be used to build an automated decision support tool to help caretakers identify at-risk patients.

## Introduction

Approximately 60% of total hip arthroplasties (THAs) and total knee arthroplasties (TKAs) are covered by Medicare nationwide. The Centers for Medicare and Medicaid Services have focused on total joint replacements as a high-volume and high-cost procedure that should be monitored for cost and quality control [1]. Therefore, bundled payment programs have been proposed to decrease the cost of procedures, shorten length of stay, and reduce the number of readmissions and revision surgeries for THAs and TKAs without sacrificing quality of care [2,3]. Accordingly, bundled payment programs penalize service providers for unscheduled or preventable readmissions [4]. In Massachusetts, for example, Medicare penalized 78% of hospitals for unscheduled readmissions between 2015 and 2016 [5]. In this case, the average penalty for hospitals was 0.7% of the Medicare reimbursement [5]. Models that can identify patients at high risk of readmission might help reduce the total costs and may also improve patient outcomes.

The increase in the use and availability of electronic health records (EHRs) has encouraged researchers to develop and evaluate predictive machine learning (ML) models exploiting EHRs. ML models built over EHRs have now been explored for many clinical predictive tasks, including diagnosis, classification, risk stratification, and medical event prediction [6-9]. A survey of this work is available in a study by Shickel et al [10].

Concerning predicting readmission, Shadmi et al [11] developed a model for 30-day readmission using manually crafted features derived from preadmission data. Similarly, Cai et al [12] used logistic regression (LR) to predict readmission and other outcomes for hospitalized patients. Nguyen et al [13] demonstrated that incorporating EHR data from the full hospital stay can improve 30-day readmission prediction, as compared with incorporating EHR data from the day of admission alone. The difference between our work and these previous efforts is that we are specifically concerned with predicting readmissions following *surgery*, rather than in general, which suggests a more focused approach and evaluation.

The idea of using ML to predict the risk of complications in patients following surgery goes back at least a few decades [14]. Recent efforts have demonstrated the general feasibility of predicting target postoperative complications [15,16]. We do not attempt to exhaustively review these efforts. To the best of our knowledge, none of these efforts have taken an exclusively data-driven approach, without the need for manual feature extraction, to predict the risk of any complications leading to readmission following hip or knee arthroplasty. We aim to address this gap in the literature. These predictions can be made passively and automatically with data from EHRs. If shown superior to direct clinical judgments, these predictions might eventually assist prioritization of proactive care and potentially mitigate complications that lead to readmissions.

This is important, partly because of the high volume of surgeries. In 2017, 700,000 knee replacement procedures were performed in the United States, and this number is likely to increase to 3.48 million surgeries by 2030 [17]. Given the rapid increase in the number of arthroplasty procedures, the need for quality and cost control in general and reducing readmissions and revision surgeries is increasingly clear. Readmissions occur for many reasons, but the 3 most common causes for readmission are surgical site infection, ileus or obstruction, and bleeding [4,17,18].

As noted above, there have been previous efforts to predict readmission risk following hip or knee surgery; however, these have relied on structured predictors manually entered by domain experts. This feature extraction process is onerous and precludes automatic and passive monitoring to identify at-risk patients. Our main contribution in this work is the development and evaluation of models for predicting postsurgery readmission directly from EHRs using unstructured clinical notes. In addition, we explored whether neural models induced over clinician notes perform as well or better than simple LR models induced over structured tabular data in the EHRs.

## Methods

### Data Set

**Figure 1:**
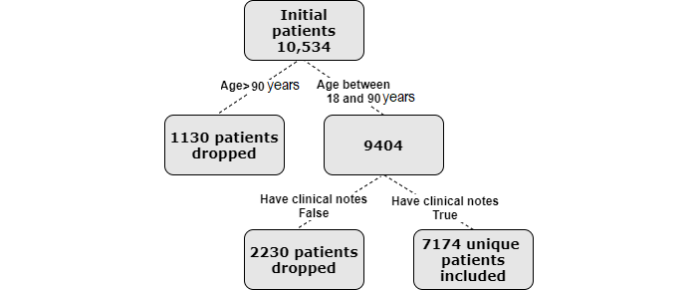
Cohort selection flow chart.

This is a retrospective analysis for which we used EHR data corresponding to 10,534 patients. We received approval from the institutional review board (protocol number 2016P002062 at Partners Healthcare) to conduct this analysis. Subjects were adults aged 18 years or older who were admitted for hip or knee surgery between 2006 and 2016 for either inpatient or outpatient care. These subjects were covered by Medicare, Medicaid, or a private insurance. Our analysis included patients who underwent hip arthroplasty (current procedural terminology [CPT] codes: 27130, 27132, 27134, 27236, 27137, 27138, 27120, and 27125) or knee arthroplasty (CPT codes: 27445, 27446, 27447, 27486, and 27487) during this period. This yielded a data set comprising 7174 patients (Figure 1).

We excluded patients who were aged above 90 years at the time of surgery because of the inherent high risk of complications [19-21], implying that no model is needed for these cases. We also excluded patients for whom no notes were present, which may have induced a sample bias, although we do not have reason to believe this is the case. Figure 1 provides a cohort selection flowchart.

### Data Types

Our models exploited (textual) clinical notes to inform predictions. We also considered the use of structured data elements within EHRs for comparison, but we encoded this automatically without domain and ML experts in the loop.

### Data Extraction and Encoding

Our primary data set consisted of clinical notes written by clinicians (doctors, nurses, and other health care professionals). These notes described patient demographics, procedures, surgeries, medications, and other medical services rendered to patients. In addition to the free text, notes sometimes contained automatically generated tables (eg, list of laboratory tests). Textbox 1 shows the EHR fields that were considered. In addition to the notes corresponding to these, we often had corresponding structured information. We describe how we preprocess this in the following section.

**Categories of features from electronic health record data used.**

Patient level:

- Demographic information
- Health history
- Health information
- Vital information
- Laboratory test results
- Comorbidities
- Medication information
- Radiology
- Procedures
- Surgical
- Pathology
- Diagnosis

Hospital level:

- Admission information

Encounter (visit)

### Structured Data Preprocessing

We extracted information pertaining to demographic, diagnostic, encounter, health history, procedures, and medications from patients’ records (Textbox 1). Patient encounters are associated with multiple diagnosis codes, including principal, secondary, and other diagnoses. We considered all diagnosis codes when determining whether a readmission was due to surgical complications. We encoded medications and diagnoses as sparse indicator vectors. To process diagnosis International Classification of Diseases (ICD) codes, we mapped ICD-9 codes to ICD-10. We retained only the first 3 ICD-10 characters to reduce sparsity.

To encode variables extracted from the health history table, we concatenated one-hot indicator vectors for all categorical features with numerical values. We encoded laboratory tests using indicator vectors that represent whether a patient received a specific test. For information pertaining to patient health history, we excluded variables that were missing from nearly all (≥99.9%) records (listed in the Multimedia Appendix 1). We also encoded patient medications using indicator vectors. We extracted admission-related information from encounter records (eg, visiting information from admission and discharge sources). For continuous variables, we replaced missing values with averages taken over all patients or encounters as appropriate. This extraction and preprocessing yields, for each patient *z*, *T* encounter records that encode structured elements 
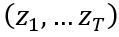
 ordered by the encounter data, where 
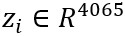
.

### Clinical Text Processing

Patients are associated with a list of free-text notes ordered by the encounter date. We tokenize them, then lowercase and stem words, which are then represented via indicator vectors (*V*). All notes are concatenated with a special delineating marker <NOTESEP>, yielding a single note of size 
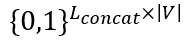
 where 
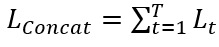
, *L_t_* represents the number of words in note for encounter *t*.

### Task Definition

We partitioned the data set at the patient level into train, validation, and test sets with a ratio of 70:15:15 (Table 1). These sets are mutually exclusive with respect to patients (ie, the same patient never appears in more than one set). Demographic statistics for training, validation, and testing sets are reported in the Multimedia Appendices 2-4, respectively. We defined the set of patients who experienced complications following surgery that led to readmission within 30 days using ICD codes. Specifically, we define this as the set of patients who underwent hip or knee surgery and who were subsequently admitted as inpatients within 30 days of their discharge under any of the ICD-9 and ICD-10 complication codes: ICD-9 codes: 996, 996 {03,1-4,57,6,66,67,7,71-73,75-79}, 997, 998 and ICD-10 codes: T84.{0X-7X, 81-86,89,9X}XA.

**Table 1:** The number of patients in training, validation, and testing data sets.

Data sets	Hip			Knee	
	Male (n=1641), n	Female (n=1658), n	Male (n=1702), n		Female (n=2173), n
Train	1131	1190	1164		1481
Validation	262	238	267		335
Test	248	230	271		357

We labeled patients who met this criterion as having been readmitted due to complications following surgery (*y*=1). We assumed that *all other patients were not readmitted due to complications* (*y*=0). There is an inherent *class imbalance* [22] here; most patients do not experience complications that lead to readmission, that is, there are far fewer positive than zero instances. We report readmission prevalence for hip and knee surgeries in Table 2.

**Table 2:** Proportion of positive class (30-day readmission because of surgery complications) for hip and knee surgeries.

Subset	Hip	Knee
Train	0.092	0.097
Validation	0.122	0.1
Test	0.115	0.116

### Models

We evaluated 2 standard neural models trained on the data set, detailed below. In addition, we implemented a simple LR model to serve as a reference.

Text is encoded into fixed-size representations for downstream modules using an *encoder*. We experimented with a few such encoders: Simple and unstructured count-based bag-of-word (BoW) representations (analogous to the indicator vectors encoding tests and medications) and neural encoders that operate over embeddings of text and learn to represent notes via repeated projection or recurrent modules.

#### Linear Models (Over Bag of Words)

For our linear model, we used *l*_1_- and *l*_2_-regularized LR over BoW representations of patient notes or the structured data associated with a given patient encounter. We considered 4 different representations of patient notes and structured data associated with a given patient encounter.

BoW variants:

- Binary BoW encodes the existence of a given word in a note as a one-hot vector.
- Count BoW encodes the total number of occurrences of a given word in a note, that is, 
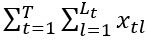
.
- Term frequency–inverse document frequency scales word counts inversely to the frequency with which they appear in documents, emphasizing comparatively rare words.
- Finally, we experimented with encoding text via inferred
  topic
  distributions using Latent Dirichlet Allocation (LDA) [23]. In this variant, we encoded texts as vectors that encode the proportions of (latent) topics present within them, as estimated via LDA. We report results for LR models that fit text and structured data.

#### Neural Encoders

Standard neural models first project words to lower-dimensional embeddings (eg, 300 dimensions initialized to pretrained embeddings). These embeddings are then passed through an encoder module before making predictions. We considered the following modules for inducing fixed-length representations of embedded variable-sized textual inputs:

1. Average: Project and then average inputs. Specifically, we first passed embeddings through a linear layer that projects them onto a 256-dimensional space and then applied an element-wise nonlinearity (ReLU).
2. Bidirectional long short-term memory (BiLSTM) network: We ran a single-layer BiLSTM [24] model over the embedded sequence using a hidden layer size of 256 (128 dimensions for each direction).

Recurrent networks (such as BiLSTM) yield variable-sized outputs that must be collapsed into a fixed-length vector. To this end, we adopted a standard max-pooling layer over the outputs of the 256 filters or hidden units. We also explored aggregation via attention mechanisms [25], which allowed models to upweight contextualized representations of specific inputs; accordingly, these have greater influence over the induced fixed-length vector. In the standard attention layer, the model learns to score each encoder hidden state *h_t_* for the input token *t* according to its relevance for the downstream prediction. Scores are normalized into a distribution *α*, and a fixed-length vector is induced by taking a weighted sum over the hidden states emitted from the RNN: 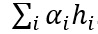 . We also explored applying attention to the feedforward (projection) encoder.

In addition, we evaluated hierarchical representation learning over clinician notes [26]. Our data contain reports from different visits. Therefore, we can consider two-level representations: visit level and patient level. An encoder can provide a representation of individual visits, and then these encoded segments can be combined (eg, via a second recurrent neural network) to form a second-level representation of the patient. The latter summarizes all visits. This is referred to as a hierarchical representation. For this, we pass a single BiLSTM to embed each patient’s notes separately (using attention), and then we run another BiLSTM over the aggregated patient-level representation of individual notes (associated with its own attention distribution) to yield a fixed-length vector.

Finally, we presented preliminary results using bidirectional encoder representations from transformers (BERT) [27] as another text encoding strategy. Specifically, we used the clinical BERT [28] instantiation of the model that was trained on clinical notes from the MIMIC III data set. BERT is a deep bidirectional model that conditions on both left and right context to provide contextualized representations of words. BERT and similar large pretrained transformer models [29] have achieved good results across many natural language processing data sets and tasks in general; specifically, they have yielded improvements for 30-day readmission tasks on the MIMIC data set [30].

### Class Imbalance

Most patients do not experience complications that result in rehospitalization within 30 days. Therefore, the resulting data sets are *imbalanced*, which can be problematic for standard ML models. We experimented with multiple strategies to counteract the class imbalance, including imposing class weights, undersampling the majority class, and oversampling the minority class. Undersampling provided consistent results across data sets and the period of history considered, whereas other strategies proved unstable.

### Multitask Learning

The most straightforward approach to predicting 30-day readmission due to complications following hip and knee arthroplasties would be to treat them as an entirely separate class of surgeries and build independent models for each type of surgery. However, intuitively one might expect the information in EHRs to be similar for complications resulting from the respective types of surgery. We can exploit this to improve predictive performance by using multitask learning [31], in which some parameters are shared between models for related tasks.

### Performance Metrics

To quantify the performance of the models in predicting 30-day readmission associated with surgical complications, we used the area under the receiver operating characteristic (AUROC) curve and accuracy, sensitivity, specificity, and precision, also known as positive predicted value (PPV), at particular thresholds. These are calculated using true positive (TP), false positive (FP), true negative (TN), and false negative (FN) as follows:

Recall (also known as sensitivity)=TP/(TP+FN)

Specificity=TN/(TN+FP)

Precision (also known as PPV)=TP/(TP+FP)

Accuracy=(TP+TN)/(TP+TN+FP+FN)

In practice, one would need to select an operational threshold with corresponding sensitivity and specificity appropriate for the intended use of the model.

To quantify model performance independent of a particular choice of threshold, we report precision versus recall, and recall versus (1-specificity) and areas underneath the corresponding curves for these constructed by sweeping thresholds (for predicting 1 vs 0) over the predicted probabilities and record corresponding metrics. The area under these can be taken as a scalar quantifying model performance.

### Experimental Setup

Before any experimentation, we separated the data into training, validation, and testing sets. The validation data were used for tuning the models and for selecting the final candidate model. The testing set was used for the final evaluation but was not used in any way during the model development and tuning.

### Data Availability

Data supporting this study are not publicly available because of the inherently sensitive nature of the data.

## Results

**Figure 2:**
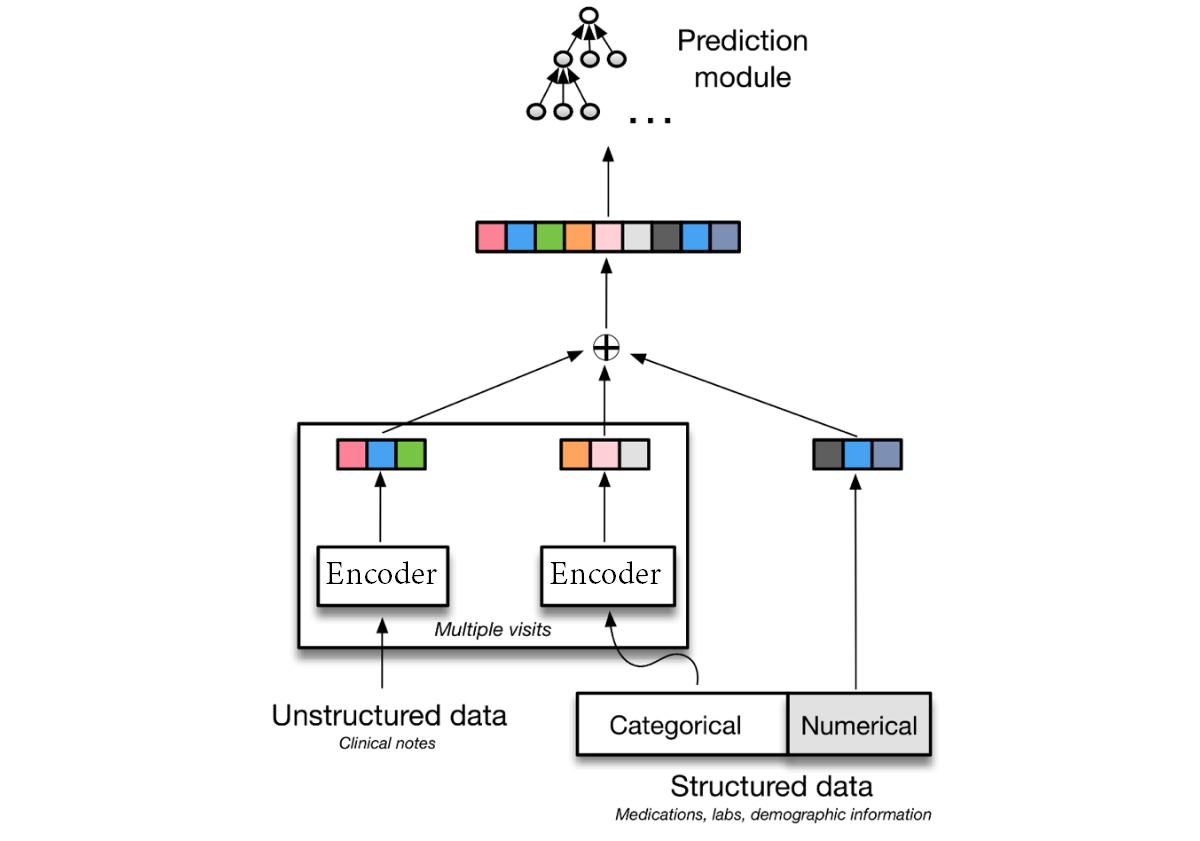
A schematic feature encoding scheme. Structured data, when used, comprises both categorical and numerical elements. We encoded the former using either indicators or an encoder module, whereas we packed the latter into a dense vector of values. Unstructured data (ie, textual notes) are encoded using a sparse (indicator) representation and then optionally run through an encoder module. Colors are stylistics only. The “+” denotes concatenation.

We tuned all hyperparameters on the validation data set. Results achieved under the best models are presented for both hip and knee surgeries as measured on the validation set for (1) text only and (2) structured data only, shown in Figure 2. The results are reported for both the validation and test data sets, where we expect better performance on the former given that we selected hyperparameters based on this. We reported the results for independent models and multitask models over text and structured data separately.

The best independent model for predicting 30-day readmission due to any complications following a hip surgery over the validation data set using text is the feedforward average model with attention mechanism (AUROC=0.894; 95% CI 0.859-0.930); for knee surgeries, the simple feedforward average model performs better (AUROC=0.946; 95% CI 0.929-0.964). Similarly, the best independent model for predicting 30-day readmission due to any complications following hip and knee surgeries using structured data is an LR model with L1 regularization with an AUROC of 0.665 (95% CI 0.589-0.732) and 0.689 (95% CI 0.630-0.749), respectively.

However, the best multitask model for predicting 30-day readmission because of any complications following a hip or knee surgery over text was a feedforward average model with an AUROC of 0.858 (95% CI 0.802-0.915) and 0.937 (95% CI 0.916-0.960), respectively. Similarly, the best multitask model trained over structured data was an LR model with L2 regularization (λ=0.001) with an AUROC of 0.676 (95% CI 0.617-0.738) following hip surgery and an AUROC of 0.664 (95% CI 0.591-0.738) following a knee surgery.

**Figure 3:**
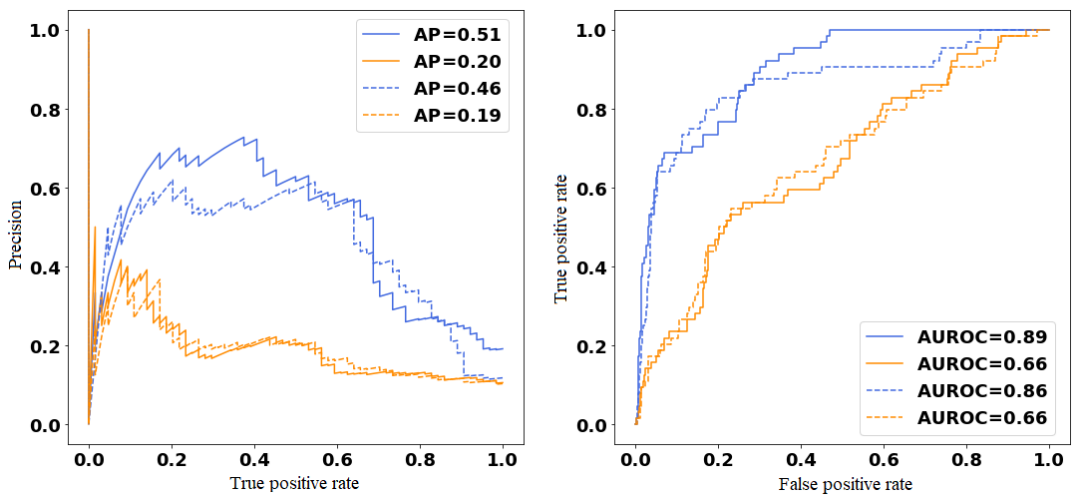
Precision-recall curve (left) and area under the receiver operating characteristic (AUROC; right) curve for hip validation set. Individual model text (blue), structured (orange), multitask models’ text (dashed blue), and structured (dashed orange).

**Figure 4:**
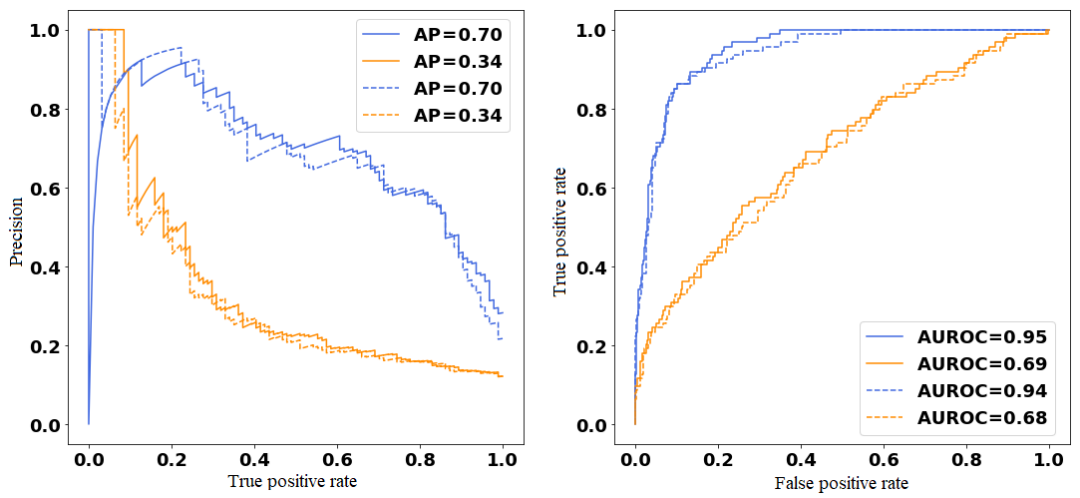
Precision-recall curve (left) and area under the receiver operating characteristic (AUROC; right) curve for knee validation set. Individual model text (blue), structured (orange), multitask models’ text (dashed blue), and structured (dashed orange).

Similarly, the BERT model for predicting 30-day readmission due to any complications following a hip or knee surgery achieved an AUROC of 0.735 (95% CI 0.701-0.785) and 0.820 (95% CI 0.782-0.843), respectively. Therefore, an independent feedforward model over text was selected as the final model to be evaluated for prediction of 30-day unplanned readmission following knee surgery. Similarly, an independent feedforward model with an attention mechanism developed over text was selected as the model to be evaluated for prediction of 30-day unplanned readmission following hip surgery (Figures 3 and 4).

We also experimented with a combination of text and structured data (Figure 2). We have not included the results of this experiment in this study because the predictive performance is worse than what we achieved using the text alone. This may seem counterintuitive, but the notes here are relatively rich in information having been manually composed to convey salient information; although these are also noisy at times. It is also entirely possible that alternative feature encodings or model architectures would result in improved model performance with structured data.

We applied the best models (as selected on the validation set) to the test set, realizing an AUROC of 0.846 (95% CI 0.823-0.871) for hip and 0.822 (95% CI 0.809-0.862) for knee surgery.

These AUROCs indicate that the model discriminates between high- and low-risk patients reasonably well. Operationally, such models might conceivably be used to rank patients with respect to their risk of requiring readmission owing to surgical complications and then to provide proactive care (presumably prioritizing limited resources) accordingly. This use would suggest capitalizing on the risk scores and corresponding rankings induced by these directly.

Alternatively, one might seek to establish a binary threshold over model outputs, indicating whether or not action needs to be taken. The appropriate threshold will depend on the intended use of such a predictive signal, which in turn would depend on the clinical actions at one’s disposal and the resources available to take these actions.

Hypothetically, we might entertain 2 settings: first, we prioritize *recall* (ie, *sensitivity*) to identify patients who will need to be readmitted without further intervention at the expense of false-positives and, second, we instead prioritize *precision* (ie, *PPV*) mindful of minimizing false-positives. These 2 settings might correspond, respectively, to a provider who has plentiful resources to provide proactive care (and so false-positives are less of a concern) and a provider who has quite limited resources, which need to be allocated carefully to mitigate false-positive cases.

With this in mind, we selected somewhat arbitrary but illustrative target metrics of 0.95 sensitivity for the former setting and 0.50 precision for the latter. We then selected corresponding thresholds on the validation data and report the results achieved using these on the test data set. Using the first (high-recall) threshold (recall=0.95; precision=0.36 on validation data), the model for readmissions due to complications following knee surgery achieved 0.79 recall and 0.27 precision on test data (classifying *everyone* as positive achieves perfect recall and 0.12 precision). The higher precision threshold (precision=0.50; recall=0.86 on validation data) yields a sensitivity of 0.70 and a precision of 0.40 on test data. For hip surgery, the results are 0.86 recall and 0.22 precision for the high-sensitivity threshold (compared with perfect recall and 0.21 precision) and 0.53 recall and 0.54 precision for the high-precision threshold. We provide results for additional thresholds in Multimedia Appendices 5 and 6.

The clinical utility of such models would, again, depend on how predictions were used in practice.

### Related Studies

Previous work has introduced models intended to predict *the risk of readmissions because of complications following colorectal, cardiac, and abdominal surgeries.* For example, Martin et al [32] evaluated predictive factors of hospital readmission rates for 266 patients undergoing abdominal surgical procedures. Wick et al [33] studied the factors associated with readmission using 7 years of data from 10,882 patients who had undergone colorectal surgery. A recent review revealed that the previous predictive models included variables such as patient comorbidities and records of previous hospitalizations [34]. A few other efforts have examined variables associated with severity of illness, laboratory tests, clinical notes from the EMR, and overall health status [33].

The American College of Surgeons National Surgical Quality Improvement Program (ACS-NSQIP) has developed a web-based surgery risk prediction tool that uses structured patient data and LR models to predict risks of complications due to surgery [35]. Edelstein et al [36] evaluated how well ACS-NSQIP can predict 30-day complications following knee and hip replacement surgeries. Mesko et al [37] identified variables predictive of readmission following hip or knee arthroplasty. These approaches rely on a small set of predefined predictors crafted by domain experts that must be manually entered for individuals.

## Discussion

Unplanned hospital readmissions impose burdens on the health care system. It is imperative for providers to improve routine follow-up protocols and provide better continuity of care with primary care physicians and other clinicians [38]. Readmission risk prediction models, such as those considered here, might provide insights that could aid decision makers in reducing rehospitalizations and readmissions by identifying patients who might be prioritized to receive proactive care.

Hospital readmissions are a key performance indicator used to measure the quality of care and cost effectiveness of the services provided. In the state of Massachusetts, TKAs and THAs corresponded to a relatively high rate of readmissions from 2010 to 2012 with 3.92% [39]. According to the Nationwide Readmission Database [40] for 224,465 patients participating in the database, the 30-day readmission rate for TKAs is between 3% and 4% depending on Medicare and non-Medicare beneficiaries. A model developed by Urish et al [41] reported that the overall median cost for each 30-day readmission was US $6753 (SD 175), constituting 36% of the overall inpatient cost for 30 days from the index procedures*, which is quite significant.* Clair et al [42] reported the average cost of readmission due to surgical complications after THA and TKA as US $22,775 and US $24,183, for a 90-day readmission with an average readmission time of 31 and 29 days, respectively. The reported costs can be decreased significantly if an appropriate prevention plan is implemented for high-risk patients that are recognized by the adoption of our modeling approach.

We have evaluated several ML algorithms that predict the risk of 30-day readmission following hip and knee arthroplasties by using real-world (unstructured and structured) EHR data obtained from the Partners Healthcare organization. On the basis of the procedure report, the proposed model is able to detect at-risk patients in cases even when there is no sign of complications immediately following the surgery. As evidence for this observation, we reproduce 2 deidentified procedure examples in the Multimedia Appendices 7 and 8.

This study has several limitations, both technical and conceptual. First, we have not evaluated the models’ *predictive performance* by comparing predictions with risk of complications of patients as assessed by surgeons or other health care personnel. This may prove to be a strong baseline, but to the best our knowledge, none of the readmission studies used this baseline. However, the fact that risk prediction tools (which rely on manual feature extraction for individual patients) have been studied extensively in this domain suggests a desire for predictive decision aids.

Second, this was a retrospective study using a convenience sample of patient EHR data, which has inherent limitations. Third, although we have demonstrated that ML models can realize reasonably strong overall discriminative performance (in terms of AUROC), translating this into a useful tool in practice would require specifying a threshold that might trigger action. We evaluated a few such hypothetical thresholds but did not have a clinical basis for these values at the time. However, it is likely that this would depend on the setting in which such models were used.

Fourth, we performed a naïve imputation for missing values, but advanced techniques, including Bayesian [43] and neural system attribution approaches [44], may improve execution. We also excluded variables with a high portion of missing values (≥99%) in patient records; according to domain experts involved in this project, a few of these excluded variables are likely to be clinically relevant. Fifth, we converted the medications and laboratory results into indicator vectors, which may result in information loss, though this was a choice made in consultation with domain experts. Sixth, we used a manually handpicked set of ICD codes to create *labels*, that is, to categorize patients as experiencing complications or not; these ICD codes may be incomplete and may introduce unknown biases in our *positive* samples. Seventh, we excluded patients aged >90 years from our analysis, as we consider such patients to be inherently at high risk.

Finally, the smaller BERT models we used are limited by the size of the document (512 words), whereas most reports here are longer than the limit. In addition, we do not have resources to pretrain BERT on our data set. That said, we tried to follow the clinical BERT methodology to make predictions at the sentence level first and then aggregate the predictions, but this approach did not perform better than the existing neural encoders on our tasks. Although we believe that a more careful application of BERT may result in improvements, it is not a straightforward task, one that needs more research and is not the main goal of the paper.

### Conclusions

We presented an ML approach to predict the risk of 30-day readmission following hip or knee arthroplasty using data directly gleaned from EHRs. Previous work on this important problem relied on manually crafted and engineered features, which neither scale nor allow automated surveillance of patients.

We found that our architecture and implementation using the text only (ie, the clinician notes) yielded predictive performance across tasks comparable with approaches using a combination of structured data and text. This suggests that the text contains rich information useful for predicting readmissions. In this case, we also found that adopting a multitask approach (sharing parameters between the models for complications following hip and knee surgeries) did not improve model performance.

We did not aim to identify the specific complication that a patient is comparatively likely to experience. Instead, we offer a patient risk stratification model intended to be used to identify high-risk patients (ie, those most likely to be readmitted) once a clinically meaningful threshold is established. Patients deemed at high risk of readmission because of complications may be scheduled for additional near-term revisits, and in general, be provided with additional proactive care and monitoring. For example, for those identified as high-risk patients, the clinic that is implementing this tool might have a nurse follow-up scheduled for the patient to ensure a continuum of care. This type of risk stratification followed by a nurse intervention in high-risk patients has been shown to produce favorable outcomes, including decreased hospitalizations and cost of care for patients regardless of the complication type [45].

We hope that this initial effort inspires additional work on automatically predicting the risk of readmission because of complications ensuing from hip and knee surgeries because such models have the potential to reduce costs and, more importantly, improve patient outcomes.

## Appendix

Multimedia Appendix 1 Variables dropped from consideration because of a high proportion of missing values (>99.9%).

Multimedia Appendix 2 Training set demographic.

Multimedia Appendix 3 Validation set demographic.

Multimedia Appendix 4 Testing set demographic.

Multimedia Appendix 5 Sample of points on validation set area under the receiver operating characteristic curve for the best model developed on knee surgery.

Multimedia Appendix 6 Sample of points on validation set area under the receiver operating characteristic curve for the best model developed on hip surgery.

Multimedia Appendix 7 Surgical texts example: surgery with complication.

Multimedia Appendix 8 Surgical texts example: surgery without complication.

## Abbreviations

ACS-NSQIP: American College of Surgeons National Surgical Quality Improvement Program
AUROC: area under the receiver operating characteristic
BERT: bidirectional encoder representations from transformers
BiLSTM: bidirectional long short-term memory
BoW: bag of words
CPT: current procedural terminology
EHR: electronic health record
FN: false negative
FP: false positive
ICD: International Classification of Diseases
LDA: Latent Dirichlet Allocation
LR: logistic regression
ML: machine learning
PPV: positive predicted value
THA: total hip arthroplasty
TKA: total knee arthroplasty
TN: true negative
TP: true positive
