# Supplementary material for: Predicting Unplanned Readmissions Following a Hip or Knee Arthroplasty: Retrospective Observational Study
Source: JMIR Med Inform. 2020 Nov 27;8(11):e19761. doi: 10.2196/19761 (PMC7732713; doi:10.2196/19761)
Supplement: Multimedia Appendix 1 [file medinform_v8i11e19761_app1.docx]

Multimedia Appendix 1. *Variables dropped from consideration because of a high proportion of missing values (>99.9%).*

| AAA (Abdominal Aortic Aneurysm) Screening , ANA screen , Albumin/creatinine ratio , Alcohol Drinks Per Week , Alcohol Oz Per Week , Alcohol Use Screening , Amino Acids , Amino Acids, urine , Antibody Screen , Antiphospholipid Antibodies , Antiphospholipid Antibody , Auto-Antibodies , B12 injection , Blood Gases/Oximetry , Blood Pressure-LFA1162 , Blood Type , Body Surface Area (BSA) , Bone Marrow Stain , Bone density , Breast Exam , Breast Exam LHA3537 , Breast Exam - LHA4003 , Breast Exam Instruction , CRYOs , CSF Chemistries , CSF Counts and Diff , CSF/Fluid, Other , Calcium Requirements Recommendation , Carnitine, serum , Carnitine, urine , Chlamydia , Cholesterol , Cholesterol-HDL , Cholesterol-LDL , Cigarettes , Coagulation Factor Studies , Colonoscopy , Complement , Complete Physical Exam , Condoms , Creatinine , Cystic Fibrosis Carrier , DNA Diagnostic Tests , DPT , DS Glucose , Dental Exams , Depo-provera Shot , Diet , Diphtheria and Tetanus booster (DT booster) , Domestic Violence Screening , Drug Use Screening , Drugs A-E , Drugs F-N , Drugs O-Z , EGD (upper GI endoscopy) , EKG , Echocardiogram , Exercise Advice , FEV1-pre (Pre-Forced Expiratory Volume) , FVC-pre (Pre-Forced Vital Capacity) , Fetal Activity , Fluid Chemistries , Fluid Counts and Diff , Folic Acid Recommendation , Foot exam , Functional Status Screen , GFR (estimated) , Glucose , Gonorrhea , HCG (Human Chorionic Gonadotropin) , HCV Ab-LHA3507 , HIVx, Haemophilus Influenzae type B (HIB) , Hand Gun Counseling , HbA1c (Hemoglobin A1c) , Hct (Hematocrit) , Head Circumference , Hearing , Hemocult x 3 , Hemoglobin Electrophoresis , Hepatitis A vaccine (Hep A vac) , Hepatitis B vaccine (Hep B vac) , Hgb (Hemoglobin) , HgbAIC , Home Hemocult , Home glucose monitoring , Hypercoagulation Studies , Hypoglycemia Assessment/Counseling , INR Result , Immune globulin , Inhibitors , Japanese encephalitis , KPS (Karnofsky performance status) , Liver - AST , Liver - Alkaline Phosphatase , Liver - Total Bilirubin , Liver ALT , Lyme , Lyme vaccine , Lymph - % Difference , Lymph - Left Arm Volume , Lymph - Right Arm Volume , Mammogram , Measles, Mumps, Rubella (MMR) , Medicare Annual Wellness Visit , Meningococcal vaccine , Microalbumin , Nutrition Referral , O2 Saturation - LFA15000 , O2 Saturation - LFA15000.1 , O2 Saturation - LFA12575 , O2 Saturation - LFA38131 , O2 Saturation - LFA38132 , O2 Saturation - LFA4826 , O2 Saturation - LFA4828 , O2 Saturation - LFA5392 , O2 Saturation - SPO2 , OPV / IPV , , ” Ophthalmology Exam , Organic Acids, urine , PSA , Pain 0-10 , Pain Assessment , Pain Scale (0-10) , Pain Score , Pap Smear , Peak Flow , Peak Flow - LHA4483 , Pelvic Exam , Personal Best Peak Flow , Platelet Aggregation , Platelet Antibodies , Pneumovax , Podiatry exam , Positive Antibody Screen , Pregnancy Weight , Prepregnancy Height , Prepregnancy Weight , Principal ICD Procedure CD , Prostate exam , Rabies , Rabies immune globulin , Rapid Strep , Rectal Exam , Rh Factor , Routine Serology , Safe Sexual Practice Counseling , Seat belt counseling , Second hand smoke exposure , Sigmoidoscopy , Smoking Quit Date , Smoking Start Date , Special Coagulation Interp , Stool Guaiac - 3 , Stool Guaiac-LHA4072 , T-cell subsets , TSH-LHA18009 , Testicular Exam , Testicular Exam Instruction , Tetanus, Diphtheria, accellular Pertussis vaccine , Tobacco Pack Per Day , Tobacco Used Years , Toxicology , Triglycerides , Trisomy 21 , Tuberculin purified protein derivative , Typhoid , UA-Protein , Urine Chemistries , Urine Chemistries Timed , Urine Chemistries Unspec , Urine Culture , Urine Dip-LHA4935 , Urine Glucose , Urine Protein , Urine Toxicology , VAS score , Varicella , Vision , Vision-Left Eye , Vision-Right Eye , Vitamin D (25 OH) , Weight Management , |
| --- |
