# Supplementary material for: Predicting Unplanned Readmissions Following a Hip or Knee Arthroplasty: Retrospective Observational Study
Source: JMIR Med Inform. 2020 Nov 27;8(11):e19761. doi: 10.2196/19761 (PMC7732713; doi:10.2196/19761)
Supplement: Multimedia Appendix 2 [file medinform_v8i11e19761_app2.docx]

Multimedia Appendix 2. Training set demographic.

| **Sex** | **Age** |  | **Race** |  |
| --- | --- | --- | --- | --- |
| Female | **Min.** | 20 | **WHITE** | 2353 |
|  | **1st Qu.** | 62 | **BLACK OR AFRICAN AMERICAN** | 107 |
|  | **Median** | 69 | **ASIAN** | 62 |
|  | **Mean** | 68.92 | **OTHER** | 179 |
|  | **3rd Qu.** | 77 | **Hispanic** | 43 |
|  | **Max.** | 89 |  |  |
| **Total** |  |  |  | **2744** |
|  |  |  |  |  |
| **Male** | **Min.** | 24 | **WHITE** | 2007 |
|  | **1st Qu.** | 59 | **BLACK OR AFRICAN AMERICAN** | 54 |
|  | **Median** | 68 | **UNKNOWN** | 109 |
|  | **Mean** | 66.82 | **Hispanic** | 25 |
|  | **3rd Qu.** | 75 | **ASIAN** | 21 |
|  | **Max.** | 89 |  |  |
| **Total** |  |  |  | **2216** |
