# Supplementary material for: Predicting Unplanned Readmissions Following a Hip or Knee Arthroplasty: Retrospective Observational Study
Source: JMIR Med Inform. 2020 Nov 27;8(11):e19761. doi: 10.2196/19761 (PMC7732713; doi:10.2196/19761)
Supplement: Multimedia Appendix 3 [file medinform_v8i11e19761_app3.docx]

Multimedia Appendix 3. Validation set demographic.

| **Sex** | **Age** |  | **Race** |  |
| --- | --- | --- | --- | --- |
| Female | **Min.** | 38 | **WHITE** | 473 |
|  | **1st Qu.** | 62 | **BLACK OR AFRICAN AMERICAN** | 25 |
|  | **Median** | 70 | **ASIAN** | 17 |
|  | **Mean** | 69.55 | **OTHER** | 34 |
|  | **3rd Qu.** | 77 | **Hispanic** | 6 |
|  | **Max.** | 89 |  |  |
| **Total** |  |  |  | **555** |
|  |  |  |  |  |
| **Male** | **Min.** | 26 | **WHITE** | 457 |
|  | **1st Qu.** | 59 | **BLACK OR AFRICAN AMERICAN** | 9 |
|  | **Median** | 67.5 | **UNKNOWN** | 29 |
|  | **Mean** | 66.54 | **Hispanic** | 7 |
|  | **3rd Qu.** | 75 | **ASIAN** | 6 |
|  | **Max.** | 89 |  |  |
| **Total** |  |  |  | **508** |
