# Supplementary material for: Predicting Unplanned Readmissions Following a Hip or Knee Arthroplasty: Retrospective Observational Study
Source: JMIR Med Inform. 2020 Nov 27;8(11):e19761. doi: 10.2196/19761 (PMC7732713; doi:10.2196/19761)
Supplement: Multimedia Appendix 4 [file medinform_v8i11e19761_app4.docx]

Multimedia Appendix 4. Testing set demographic.

| **Sex** | **Age** |  | **Race** |  |
| --- | --- | --- | --- | --- |
| Female | **Min.** | 25 | **WHITE** | 481 |
|  | **1st Qu.** | 62 | **BLACK OR AFRICAN AMERICAN** | 31 |
|  | **Median** | 69 | **ASIAN** | 10 |
|  | **Mean** | 69.12 | **OTHER** | 30 |
|  | **3rd Qu.** | 77 | **Hispanic** | 11 |
|  | **Max.** | 89 |  |  |
| **Total** |  |  |  | **563** |
|  |  |  |  |  |
| **Male** | **Min.** | 28 | **WHITE** | 448 |
|  | **1st Qu.** | 60 | **BLACK OR AFRICAN AMERICAN** | 16 |
|  | **Median** | 68 | **UNKNOWN** | 28 |
|  | **Mean** | 67.09 | **Hispanic** | 6 |
|  | **3rd Qu.** | 75 | **ASIAN** | 6 |
|  | **Max.** | 89 |  |  |
| **Total** |  |  |  | **501** |
