# Supplementary material for: Predicting Unplanned Readmissions Following a Hip or Knee Arthroplasty: Retrospective Observational Study
Source: JMIR Med Inform. 2020 Nov 27;8(11):e19761. doi: 10.2196/19761 (PMC7732713; doi:10.2196/19761)
Supplement: Multimedia Appendix 5 [file medinform_v8i11e19761_app5.docx]

Multimedia Appendix 5. Sample of points on validation set area under the receiver operating characteristic curve for the best model developed on knee surgery*.*

| **threshold** | **specificity** | **sensitivity** | **accuracy** | **tn** | **tp** | **fn** | **fp** | **npv** | **ppv** | **1-accuracy** | **1-npv** | **1-ppv** |
| --- | --- | --- | --- | --- | --- | --- | --- | --- | --- | --- | --- | --- |
| 0.2170 | 64.9560 | 100.0000 | 69.2010 | 443 | 94 | 0 | 239 | 100.000 | 28.228 | 30.799 | 0.000 | 71.772 |
| 0.2173 | 64.9560 | 98.9362 | 69.0722 | 443 | 93 | 1 | 239 | 99.775 | 28.012 | 30.928 | 0.225 | 71.988 |
| 0.2179 | 65.1026 | 98.9362 | 69.2010 | 444 | 93 | 1 | 238 | 99.775 | 28.097 | 30.799 | 0.225 | 71.903 |
| 0.2182 | 65.2493 | 98.9362 | 69.3299 | 445 | 93 | 1 | 237 | 99.776 | 28.182 | 30.670 | 0.224 | 71.818 |
| 0.2190 | 65.3959 | 98.9362 | 69.4588 | 446 | 93 | 1 | 236 | 99.776 | 28.267 | 30.541 | 0.224 | 71.733 |
| 0.2198 | 65.5425 | 98.9362 | 69.5876 | 447 | 93 | 1 | 235 | 99.777 | 28.354 | 30.412 | 0.223 | 71.646 |
| 0.2199 | 65.6891 | 98.9362 | 69.7165 | 448 | 93 | 1 | 234 | 99.777 | 28.440 | 30.284 | 0.223 | 71.560 |
| 0.2199 | 65.8358 | 98.9362 | 69.8454 | 449 | 93 | 1 | 233 | 99.778 | 28.528 | 30.155 | 0.222 | 71.472 |
| 0.2200 | 65.9824 | 98.9362 | 69.9742 | 450 | 93 | 1 | 232 | 99.778 | 28.615 | 30.026 | 0.222 | 71.385 |
| 0.2204 | 66.1290 | 98.9362 | 70.1031 | 451 | 93 | 1 | 231 | 99.779 | 28.704 | 29.897 | 0.221 | 71.296 |
| 0.2210 | 66.2757 | 98.9362 | 70.2320 | 452 | 93 | 1 | 230 | 99.779 | 28.793 | 29.768 | 0.221 | 71.207 |
| 0.2217 | 66.4223 | 98.9362 | 70.3608 | 453 | 93 | 1 | 229 | 99.780 | 28.882 | 29.639 | 0.220 | 71.118 |
| 0.2221 | 66.5689 | 98.9362 | 70.4897 | 454 | 93 | 1 | 228 | 99.780 | 28.972 | 29.510 | 0.220 | 71.028 |
| 0.2223 | 66.7155 | 98.9362 | 70.6186 | 455 | 93 | 1 | 227 | 99.781 | 29.063 | 29.381 | 0.219 | 70.938 |
| 0.2227 | 66.8622 | 98.9362 | 70.7474 | 456 | 93 | 1 | 226 | 99.781 | 29.154 | 29.253 | 0.219 | 70.846 |
| 0.2230 | 67.0088 | 98.9362 | 70.8763 | 457 | 93 | 1 | 225 | 99.782 | 29.245 | 29.124 | 0.218 | 70.755 |
| 0.2231 | 67.1554 | 98.9362 | 71.0052 | 458 | 93 | 1 | 224 | 99.782 | 29.338 | 28.995 | 0.218 | 70.662 |
| 0.2232 | 67.3021 | 98.9362 | 71.1340 | 459 | 93 | 1 | 223 | 99.783 | 29.430 | 28.866 | 0.217 | 70.570 |
| 0.2233 | 67.4487 | 98.9362 | 71.2629 | 460 | 93 | 1 | 222 | 99.783 | 29.524 | 28.737 | 0.217 | 70.476 |
| 0.2235 | 67.4487 | 97.8723 | 71.1340 | 460 | 92 | 2 | 222 | 99.567 | 29.299 | 28.866 | 0.433 | 70.701 |
| 0.2240 | 67.5953 | 97.8723 | 71.2629 | 461 | 92 | 2 | 221 | 99.568 | 29.393 | 28.737 | 0.432 | 70.607 |
| 0.2245 | 67.7419 | 97.8723 | 71.3918 | 462 | 92 | 2 | 220 | 99.569 | 29.487 | 28.608 | 0.431 | 70.513 |
| 0.2246 | 67.8886 | 97.8723 | 71.5206 | 463 | 92 | 2 | 219 | 99.570 | 29.582 | 28.479 | 0.430 | 70.418 |
| 0.2251 | 68.0352 | 97.8723 | 71.6495 | 464 | 92 | 2 | 218 | 99.571 | 29.677 | 28.351 | 0.429 | 70.323 |
| 0.2262 | 68.1818 | 97.8723 | 71.7784 | 465 | 92 | 2 | 217 | 99.572 | 29.773 | 28.222 | 0.428 | 70.227 |
