# Supplementary material for: Predicting Unplanned Readmissions Following a Hip or Knee Arthroplasty: Retrospective Observational Study
Source: JMIR Med Inform. 2020 Nov 27;8(11):e19761. doi: 10.2196/19761 (PMC7732713; doi:10.2196/19761)
Supplement: Multimedia Appendix 6 [file medinform_v8i11e19761_app6.docx]

Multimedia Appendix 6. Sample of points on validation set area under the receiver operating characteristic curve for the best model developed on hip surgery.

| **threshold** | **specificity** | **sensitivity** | **accuracy** | **tn** | **tp** | **fn** | **fp** | **npv** | **ppv** | **1-accuracy** | **1-npv** | **1-ppv** |
| --- | --- | --- | --- | --- | --- | --- | --- | --- | --- | --- | --- | --- |
| 0.0991 | 56.4236 | 95 | 60.313 | 325 | 61 | 3 | 251 | 99.085 | 19.551 | 39.688 | 0.915 | 80.449 |
| 0.0997 | 56.5972 | 95 | 60.469 | 326 | 61 | 3 | 250 | 99.088 | 19.614 | 39.531 | 0.912 | 80.386 |
| 0.1004 | 56.7708 | 95 | 60.625 | 327 | 61 | 3 | 249 | 99.091 | 19.677 | 39.375 | 0.909 | 80.323 |
| 0.1008 | 56.9444 | 95 | 60.781 | 328 | 61 | 3 | 248 | 99.094 | 19.741 | 39.219 | 0.906 | 80.259 |
| 0.1017 | 57.1181 | 95 | 60.938 | 329 | 61 | 3 | 247 | 99.096 | 19.805 | 39.063 | 0.904 | 80.195 |
| 0.1023 | 57.2917 | 95 | 61.094 | 330 | 61 | 3 | 246 | 99.099 | 19.870 | 38.906 | 0.901 | 80.130 |
| 0.1025 | 57.4653 | 95 | 61.250 | 331 | 61 | 3 | 245 | 99.102 | 19.935 | 38.750 | 0.898 | 80.065 |
| 0.1027 | 57.6389 | 95 | 61.406 | 332 | 61 | 3 | 244 | 99.104 | 20.000 | 38.594 | 0.896 | 80.000 |
| 0.1029 | 57.8125 | 95 | 61.563 | 333 | 61 | 3 | 243 | 99.107 | 20.066 | 38.438 | 0.893 | 79.934 |
| 0.1034 | 57.9861 | 95 | 61.719 | 334 | 61 | 3 | 242 | 99.110 | 20.132 | 38.281 | 0.890 | 79.868 |
| 0.1042 | 58.1597 | 95 | 61.875 | 335 | 61 | 3 | 241 | 99.112 | 20.199 | 38.125 | 0.888 | 79.801 |
| 0.1049 | 58.3333 | 95 | 62.031 | 336 | 61 | 3 | 240 | 99.115 | 20.266 | 37.969 | 0.885 | 79.734 |
| 0.1054 | 58.5069 | 95 | 62.188 | 337 | 61 | 3 | 239 | 99.118 | 20.333 | 37.813 | 0.882 | 79.667 |
| 0.1061 | 58.6806 | 95 | 62.344 | 338 | 61 | 3 | 238 | 99.120 | 20.401 | 37.656 | 0.880 | 79.599 |
| 0.1064 | 58.8542 | 95 | 62.500 | 339 | 61 | 3 | 237 | 99.123 | 20.470 | 37.500 | 0.877 | 79.530 |
| 0.1077 | 59.0278 | 95 | 62.656 | 340 | 61 | 3 | 236 | 99.125 | 20.539 | 37.344 | 0.875 | 79.461 |
| 0.1091 | 59.2014 | 95 | 62.813 | 341 | 61 | 3 | 235 | 99.128 | 20.608 | 37.188 | 0.872 | 79.392 |
| 0.1096 | 59.3750 | 95 | 62.969 | 342 | 61 | 3 | 234 | 99.130 | 20.678 | 37.031 | 0.870 | 79.322 |
| 0.1101 | 59.5486 | 95 | 63.125 | 343 | 61 | 3 | 233 | 99.133 | 20.748 | 36.875 | 0.867 | 79.252 |
| 0.1110 | 59.7222 | 95 | 63.281 | 344 | 61 | 3 | 232 | 99.135 | 20.819 | 36.719 | 0.865 | 79.181 |
| 0.1123 | 59.8958 | 95 | 63.438 | 345 | 61 | 3 | 231 | 99.138 | 20.890 | 36.563 | 0.862 | 79.110 |
| 0.1136 | 60.0694 | 95 | 63.594 | 346 | 61 | 3 | 230 | 99.140 | 20.962 | 36.406 | 0.860 | 79.038 |
| 0.1146 | 60.2431 | 95 | 63.750 | 347 | 61 | 3 | 229 | 99.143 | 21.034 | 36.250 | 0.857 | 78.966 |
| 0.1154 | 60.4167 | 95 | 63.906 | 348 | 61 | 3 | 228 | 99.145 | 21.107 | 36.094 | 0.855 | 78.893 |
