# Supplementary material for: Predicting Unplanned Readmissions Following a Hip or Knee Arthroplasty: Retrospective Observational Study
Source: JMIR Med Inform. 2020 Nov 27;8(11):e19761. doi: 10.2196/19761 (PMC7732713; doi:10.2196/19761)
Supplement: Multimedia Appendix 7 [file medinform_v8i11e19761_app7.docx]

Multimedia Appendix 7. Surgical Texts Example: Surgery with Complication.

| NAME OF OPERATION: Revision, left total knee replacement.  ANESTHESIA: General. TOURNIQUET  TIME: Approximately 80 minutes at 300 mmHg pressure.  DESCRIPTION OF PROCEDURE:  Under satisfactory anesthesia, prophylactic IV antibiotics were given. Sterile prep and drape of the left leg was done in standard manner. The leg was exsanguinated with an Esmarch bandage, and tourniquet around the thigh was inflated to 300 mmHg pressure through the time of bandage application. Anterior approach was performed with a medial parapatellar arthrotomy. There was clear fluid and no clinical sign of infection. The knee had abundant synovitis consistent with third body particle wear. Synovectomy was performed as part of the exposure. The size of the leg and tightness of the extensor mechanism made it difficult to evert the patella, and we performed a quad snip. We mobilized the patella out of the way. The patella and tibia were still solidly fixed to bone. We lifted the rotating platform tray out of the tibia, and noted multiple circumferential wear lines in it. The femoral component was noted to have early loosening with lysis underneath. We were able to easily extract the femoral component. It was posterior stabilized [**Last Name (un) 10**] Sigma PS implant. We then reamed the femoral canal for 120 mm depth up to 14 mm. We did a slight 1-2 mm trim of the distal femur. We placed the 4-in-1 cutting block onto the end of the reamer and set the rotation in a gap balance technique. Minimal additional trims were done for the lateral chamfer cut and anterior cut. Posteriorly, we cut through the 4 mm slots. We placed the final jig and recut the housing. We assembled the trial femoral component with a posterior stabilized 2.5 bearing surface, two, 4 mm posterior augments, a 2-mm offset bolt, a 5-degree adapter, and a 115 x 14 mm fluted stemextension. This was impacted in place and was an excellent fit. We placed a 12.5 thick RP-PS insert. The knee came to full extension, flexed well, was well aligned and was stable throughout range of motion. The trials were removed. Bone surfaces were cleaned with pulse lavage and dried. A few sclerotic areas were drilled for cement interdigitation. The permanent implant components were brought on the operating field and assembled. There were all of same size as noted with the trial. Bone surfaces were dried while cement was mixed. The component was cemented on the underside of the articular component and up the femoral rod just to the flutes. The real component was impacted into place. Excess cement was removed. A 2.5 PS tibial insert with a thickness of 12.5 mm was dropped into the RP tray. Final washout was done. Closure was done with #5 Ethibond to the snip area, #1 Vicryl to the capsule, 2-0 Vicryl to the subcutaneous layer and staples to the skin. Sterile dressing was applied. Tourniquet was deflated. The anesthesia was discontinued. She was taken to the Recovery Room in satisfactory condition. Dr. [**Last Name (STitle) **] was present for the entire surgical procedure. There were no known intraoperative complications. |
| --- |
