# Supplementary material for: Predicting Unplanned Readmissions Following a Hip or Knee Arthroplasty: Retrospective Observational Study
Source: JMIR Med Inform. 2020 Nov 27;8(11):e19761. doi: 10.2196/19761 (PMC7732713; doi:10.2196/19761)
Supplement: Multimedia Appendix 8 [file medinform_v8i11e19761_app8.docx]

Multimedia Appendix 8. Surgical Texts Example: Surgery without Complication.

| SPECIMEN: Polyethylene liner to [**Male First Name (un) 7**]  Orthopedic Biomechanics Lab.  ESTIMATED BLOOD LOSS: Minimal.  DESCRIPTION OF PROCEDURE:  [**First Name8 (NamePattern2) **] [**Last Name (NamePattern1) **] is brought to the Operating Room for surgical treatment of an unstable primary left total knee replacement. His knee was balanced; however, he had a feeling of lack of stability. We reviewed the various treatment options including liner exchange, total knee revision and he had strongly preferred liner exchange if at all possible. Informed consent was obtained and anesthetic was induced and he had received preoperative intravenous antibiotics. Preoperative evaluation was negative for any concern about infection. I prepped and draped his left leg and after a timeout procedure, used a portion of his previous incision and made a medial arthrotomy without entering the quadriceps above the patella. I was able to examine his knee, which showed varus valgus laxity and a bit of anterior posterior stability, removed the polyethylene trial and placed the new thicker one by a couple of millimeters. Interestingly this remarkably changed the varus valgus and AP stability, and I felt it was adequate without going to a 13. Femoral and tibial alignments were fine. Patellar tracking was good. I then placed the size 4 CS poly size 11 mm and confirmed it locked into place. He had preserved full extension and full flexion with markedly improved stability. The pneumo- tourniquet was deflated and hemostasis was obtained. I never used any retractors other than a Z retractor and small rake. The tibia did not need to be subluxed. We closed in layers and a Hemovac drain was not necessary. Ropivacaine multimodal was placed throughout the soft tissues. He was awakened, extubated and taken to recovery stable. Post total knee revision range of motion was 0 to 140 degrees with improved stability.  Dr. [**Last Name (STitle) 5**] is dictating. I was present for and performed this procedure.  Dr. [**Last Name (STitle) 6**] was the first assistant for the procedure and there were no qualified residents to assist us today. |
| --- |
